# T2-weighted cardiac magnetic resonance imaging: a quantitative approach for measuring myocardial edema after reperfusion and its persistency in acute ischemic heart disease

**DOI:** 10.1186/1532-429X-13-S1-P171

**Published:** 2011-02-02

**Authors:** Luis R Soenksen, Aloha Meave, Gabriela Meléndez-Ramirez

**Affiliations:** 1 Instituto Nacional de Cardiología - Ignacio Chávez, Mexico City, Mexico

## Background

Within the extensive range of medical imaging methods used for cardiac analysis, T2-Weighed (T2W) Cardiac Magnetic Resonance (CMR) has been one of the most promising techniques for non-invasive edema quantification and IHD severity assessment. Many authors have suggested the importance of edema quantification in the prognosis of IHD patients and in the evaluation of expected ventricular function improvement after cursory percutaneous angioplasties (PTA)

## Purpose

The aim of the present study is to determine the prognostic value of T2W-Turbo Spin Eco (T2W-TSE) when assessing presence and persistency of myocardial edema in order to obtain concrete outcome expectancies for IHD cases after percutaneous coronary intervention (PCI).

## Methods

The present is a prospective observational study in which 66 patients were analyzed with axial T2W dark blood CMR sequences for myocardial edema quantification within the first 7 days after reperfusion (<7days), obtaining a follow-up study 90 ± 4 days later. Two experienced observers with extensive knowledge in CMR imaging analysis (2-5 years; LS, GM) interpreted the studies focusing on the detection of myocardial infarct with edema, confirming the quantitative data obtained by the software cmr42. A paired 2-tailed Student's t-test was done for obtaining the correlation between myocardial salvage and theLVEF% ventricular function variable between the two observations.

## Results

All T2W MR studies were considered readable and suited for diagnostic purposes. The evaluated patients were: 60 men (90.9%) and 6 women (9.1%) of 52 ± 12 years. For the group, a reduction in myocardial percentage edema at the follow up study does not predicts an improvement in ventricular function (positive LVEF%); moreover, neither the individuals with complete edema clearance, nor the subjects where edema persisted had a significant tendency of improving ventricular function after PCI. Therefore, IHD prognosis is likely to be more related to variables like infarct size, microvascular obstruction presence than it is to edema persistency or clearance per se.

## Conclusions

Among IHD patients, edema quantification (alone) does not predicts for a significant increases of the ventricular function after coronary reperfusion. Nonetheless, T2W MRI is still a valuable tool for quantifying myocardial salvage when used in parallel to LGE sequences for infarct measurements. Infarct size and microvascular obstruction quantification in these terms are of great interest for complementing the generated data related to edema persistency and overall cardiac function.

